# TTN mutations predict a poor prognosis in patients with thyroid cancer

**DOI:** 10.1042/BSR20221168

**Published:** 2022-07-22

**Authors:** Xiudan Han, Jianrong Chen, Jiao Wang, Jixiong Xu, Ying Liu

**Affiliations:** Department of Endocrinology and Metabolism, First Affiliated Hospital of Nanchang University, Nanchang, People’s Republic of China

**Keywords:** bioinformatics analysis, gene mutations, poor prognosis, thyroid cancer, TTN gene

## Abstract

**Objective**: We aimed to investigate the relationship between titin (TTN) gene mutations and thyroid cancer (THCA) and to explore the feasibility of the TTN gene as a potential prognostic indicator of THCA.

**Methods**: From TCGA-THCA cohort, we performed a series of analyses to evaluate the prognostic value and potential mechanism of TTN in THCA. These patients were divided into the mutant-type (MUT) group and the wild-type (WT) group. Differentially expressed genes (DEGs) in the two groups were screened using the ‘DESeq2’ R package. Functional enrichment analysis was performed, and the protein–protein interaction (PPI) network, transcription factor (TF)-target interaction networks, and competitive endogenous RNA (ceRNA) regulatory networks were established for the DEGs. The TIMER database was applied for immune cell infiltration. Survival analysis and Cox regression analysis were used to analyze the potential prognostic value of the TTN gene.

**Results**: Differential expression analysis showed that 409 genes were significantly up-regulated and 36 genes were down-regulated. Functional enrichment analysis revealed that TTN gene mutations played a potential role in the development of THCA. Analysis of the immune microenvironment indicated that TTN gene mutations were significantly associated with enrichment of M0 macrophages. Survival analysis showed that the MUT group predicted poorer prognosis than the WT group. Cox regression analysis demonstrated that TTN gene mutations were an independent risk factor for THCA. Nomograms also confirmed the prognostic values of the TTN gene in THCA.

**Conclusions** In summary, our results demonstrated that TTN gene mutations predict poor prognosis in patients with THCA. This is the first study to research TTN gene mutations in THCA and to investigate their prognostic value in THCA.

## Introduction

Thyroid cancer (THCA) is the most common endocrine malignancy, and its incidence is increasing rapidly worldwide [1]. Implementation of conservative diagnostic practices did not result in a decline in THCA incidence in men [2]. The follicular-derived thyroid cancers are divided into well-differentiated papillary thyroid cancer (PTC), follicular thyroid cancer (FTC), poorly differentiated thyroid cancer (PDTC), and anaplastic thyroid cancer (ATC). PTC is one of the most common forms of THCA [3]. Although well-differentiated thyroid cancers, such as PTC, have a good prognosis, the mortality rates of PDTC and ATC are still relatively high [4]. To date, the standard treatment for THCA includes surgery, radioactive iodine (RAI) therapy, and thyroid-stimulating hormone (TSH) inhibition. However, many patients still show recurrence or progression. Notably, as more gene mutations are being recognized and becoming drug targets, more novel treatments are being studied in THCA, including the BRAF inhibitors, vemurafenib [5] and dabrafenib [6]. Although the role of gene mutations in THCA has been partially clarified, there are no clear drugs for targeted therapy, and the pathogenesis and molecular mechanisms of THCA remain to be elucidated. The treatment of refractory thyroid cancer also remains a major challenge, and intensive efforts have been made to find an effective treatment for refractory thyroid cancer [7]. Thus, the identification of new potential biomarkers to guide targeted clinical therapy is urgently needed.

In recent years, with the rapid development of molecular biology, it has been found that the occurrence and development of cancer are related to gene mutation and methylation. Many genes and biomarkers related to THCA have been reported. Currently, BRAF mutation, NTRK3 fusion, NRAS mutation, and RET fusion are of concern [8,9]. The discovery of new biomarkers has significantly improved the understanding of the molecular pathogenesis of THCA, thus providing more personalized treatment for patients with THCA. Despite significant advances in molecular therapy, there is still a lack of effective therapies for advanced and radioactive iodine-refractory differentiated thyroid cancer [10]. Good diagnostic and prognostic biomarkers still have not been found.

Studies have shown that titin (TTN) gene mutations are related to the occurrence and development of tumors. TTN genes, also known as TMD, CMH9, CMD1G, CMPD4, and EOMFC, encode proteins in striated muscle. The 127 TTN-coding sequence variants associated with human conditions are distributed as follows: 33 nonsense mutations, 32 frameshifts, 36 missense variants, and 22 splice-site variants [11]. Recent studies have indicated that mutations in the TTN gene are among the most common genomic aberrations in ocular surface squamous neoplasia (OSSN) [12]. TTN was identified as the most frequently mutated gene within the pancancer cohort, and its mutation number was best correlated with tumor mutation burden (TMB) [13]. Yang et al. found that TTN mutations were associated with prognosis and higher TMB in gastric cancer [14]. This evidence supports the key role of the TTN gene in the carcinogenesis process. However, no studies have reported the relationship between TTN gene mutations and THCA. The present study focused on TTN gene mutations in THCA and explored the molecular mechanisms related to TTN gene mutations through bioinformatics analysis, which has not yet been reported.

In the present study, we aimed to explore the relationship between TTN gene mutations and THCA and to explore the feasibility of the TTN gene as a potential prognostic indicator of THCA. The results suggested that TTN gene mutations were closely related to patients with THCA and that the TTN gene was a potential clinical biomarker of THCA.

## Methods

### Data sources

‘Masked Somatic Mutation’ data can be obtained from the TCGA GDC website and was defined as somatic mutation data in patients with THCA in the present study. Data were processed using VarScan software, and visualization of somatic mutations was performed using the MAFTools R package [15]. Gene expression sequencing data (HTseq counts) of patients with THCA (*n*=568) were downloaded. In addition, clinical data of the control group (*n*=507) were downloaded using TCGA GDC software, including age, survival status, follow-up time, clinical stage, etc. Due to the exclusion of data with no survival information and incomplete TNM-staging information, 495 patients were retained for further analysis of clinical baseline data. These data were divided into the TTN gene mutation (mutant-type, MUT) group and the wild-type (WT) group.

### Copy number variation analysis

To analyze the change in TTN gene copy-number variations in TCGA-THCA, the ‘TCGAbiolinks’ R package [16] was used to download the ‘Masked Copy Number Segment’ data. Copy number variations (CNVs) between the MUT group and the WT group were explored by GISTIC 2.0 [17] analysis of gene copy numbers from GenePattern (https://cloud.genepattern.org) [18].

The total number of somatic mutations detected in the tumor was defined as the TMB [19]. The Wilcoxon rank-sum test was applied to compare the overall difference in the level of TMB between the two groups.

### Gene expression analysis

Differential gene expression refers to alterations in the expression (counts) of the sum of each of the isoforms that are encoded by a gene. The Wilcoxon rank-sum test was used to compare gene expression levels. In addition, differentially expressed genes (DEGs) in the two groups were screened by the ‘DESeq2’ R package [20]. The results are presented in the form of heatmaps and volcanoes. Adjusted *P*<0.05 and |log2fold change (FC)| > 1 were chosen as the cutoff criteria.

### Gene set enrichment analysis

Gene set enrichment analysis (GSEA) is designed to analyze the association between gene sets and biological signals in one dataset [21]. To study the differences in biological processes between the two groups, GSEA was performed. The ‘c2.cp.v7.2.symbols [Curated]’ gene set was downloaded from the MSigDB database [22] for GSEA. Then, genetic ontology (GO) terms and Kyoto Encyclopedia of Genes and Genomes (KEGG) pathways were analyzed. GO analysis is a powerful bioinformatics tool used to identify biological processes (BPs), cellular components (CCs), and molecular functions (MFs) [23]. KEGG is a widely used database that stores information about genomes, biological pathways, diseases, and drugs [24]. The ‘ClusterProfiler’ R package [25] was used for GO enrichment analysis and KEGG pathway enrichment analysis. Adjusted *P*<0.05 and false discovery rate (FDR) < 0.25 were considered statistically significant.

### Protein–protein interaction and hub gene identification

Protein–protein interaction (PPI) information was generated using STRING, which is an online database that identifies functional associations between proteins [26]. Genes (score > 0.9) were selected to construct the network model, and the results were visualized by Cytoscape (V3.7.2) software. Then, the maximal clique centrality (MCC) of the node genes in the PPI network was calculated by CytoHubba [27], and the top ten genes were selected as hub genes. Furthermore, we predicted the transcription factor (TF) and competitive endogenous RNA (ceRNA) networks interacting with DEGs using the Cistrome database (http://www.cistrome.org/) [28] and miRTarBase database (https://mirtarbase.cuhk.edu.cn/) [29], respectively. The corresponding interactive diagrams were generated using Cytoscape software.

### Immune cell infiltration

CIBERSORT was used to quantify the abundance of immune cells in THCA samples, and the LM22 (10 of 22 cell subsets) signature was performed to distinguish 22 phenotypes of human-infiltrating immune cells [30]. To quantify the proportion of different immune cells in the THCA sample, we used the TIMER database to analyze the correlation between TTN gene mutations and immune cell infiltration in the TCGA-THCA dataset. The TIMER database [31], a web server for the comprehensive analysis of tumor-infiltrating immune cells, provides a user-friendly web interface for dynamic analysis and visualization.

### Construction and validation of the clinical prediction model

Survival analysis was performed in both the MUT group and the WT group using the ‘survival’ R package [32]. To further investigate the influence of TTN gene mutations combined with clinicopathological features on the prognosis of patients, univariate and multivariate Cox regression analyses were applied to analyze the independent predictive ability of the risk score for patients with THCA. Subsequently, the TTN gene mutations and clinical information in the TCGA-THCA dataset were used to construct a nomogram that can predict the prognosis of THCA.

### TTN gene mutations and drug sensitivity

The Genomics of Drug Sensitivity in Cancer (GDSC) database (http://www.cancerRxgene.org) is the largest public resource for information on drug sensitivity in cancer cells and molecular markers of drug response [33]. The PRRophetic algorithm [34] was used to construct a regression model based on the expression profile of the GDSC cell line and TCGA-THCA gene. IC50 analysis was used to predict the sensitivity of THCA samples to anticancer drugs.

### Statistical analysis

Data processing and analysis were mostly achieved using R language, version 4.0.2. Most of the statistical analyses were performed using the bioinformatic tools mentioned above. IBM SPSS statistics 26.0 software was utilized for statistical analysis, and *P*<0.05 was considered to indicate a statistically significant difference.

## Results

### Analysis of mutation level and chromosome CNV in patients with THCA

**Figure 1:**
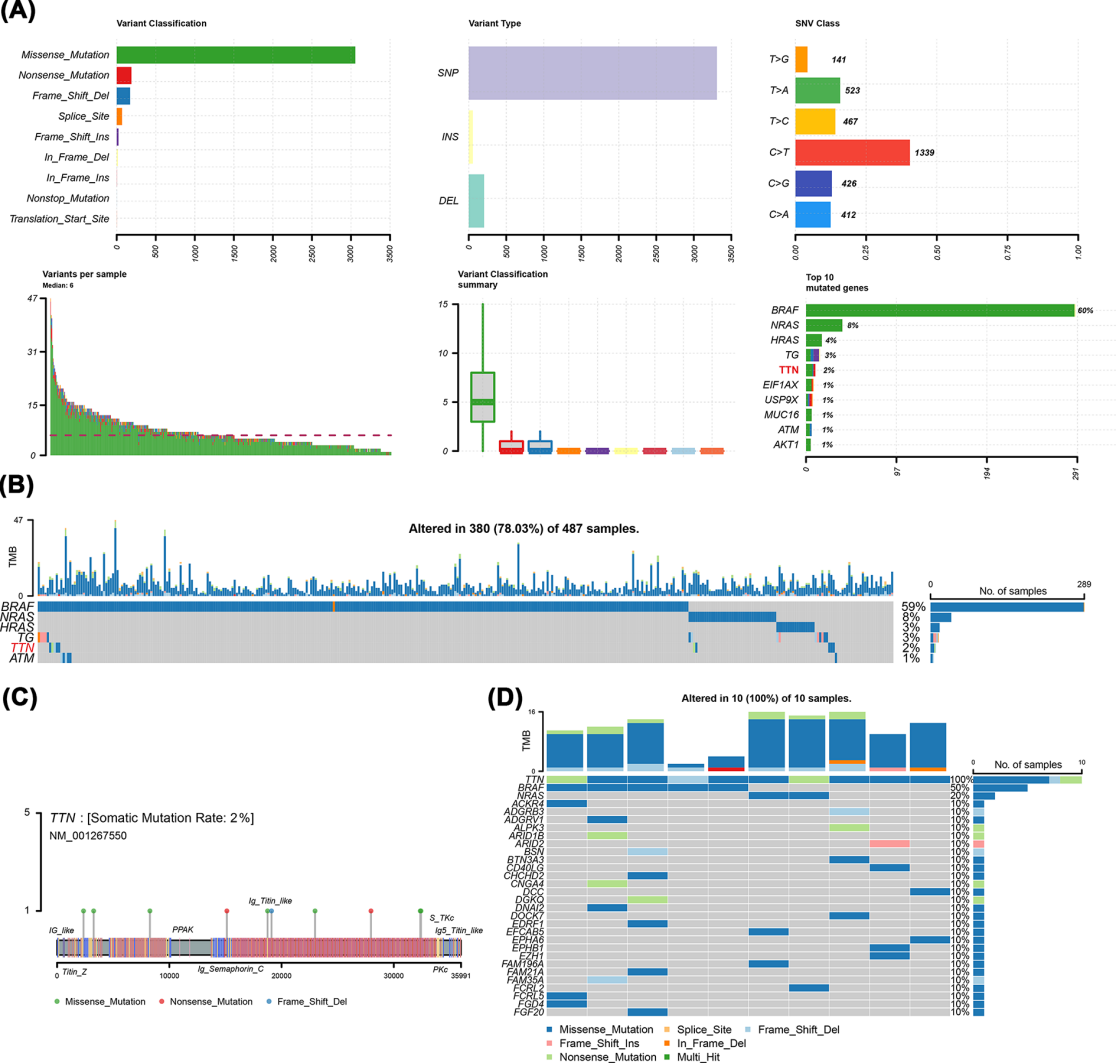
Analysis of the level of TTN mutations in patients with THCA (**A**) Panoramic images of gene mutations. (**B**) The top five mutated genes in the TCGA-THCA dataset. (**C**) Amino acid changes of TTN protein in TCGA-THCA dataset. (**D**) Analysis of overall mutation signature in the MUT group.

To analyze the impact of gene mutations on THCA, the characteristics of 495 patients with THCA including clinical and gene mutation data were collected from TCGA database. The baseline characteristics of the TCGA-THCA cohort are summarized in Table 1. The results showed that missense mutation was the main mutation type and the frequency of single-nucleotide polymorphisms was higher than that of insertions or deletions, and C>T was most common in single-nucleotide variants (SNVs) in THCA (Figure 1A). Furthermore, among all patients with THCA, the top five mutated genes were BRAF, NRAS, HRAS, TTN, and TG (Figure 1B). In recent years, there have been many reports on the study of BRAF and RAS gene mutations in THCA [35–37], but the role and mechanism of TTN gene mutations in THCA are not clear, and TTN gene mutations in THCA are rarely studied. Consequently, the present study focused on TTN gene mutations in THCA and thoroughly explored TTN mutation-related genes and potential mechanisms through bioinformatics methods. According to the mutation sites, we plotted the mutational landscapes of the TTN gene (Figure 1C). We further made the mutation panorama using the mafTools package (Figure 1D). In addition, we analyzed CNV differences between the two groups. The results indicated that compared with the WT group, the MUT group did not have a significant copy number amplification but did have a significant copy number deletion (Supplementary Figure S1).

**Table 1:** The baseline characteristics of the TCGA-THCA cohort

Characteristics	N (%)
**Gender**	
Female	361 (73)
Male	134 (27)
**Age**	
<60	378 (76)
≥60	117 (24)
**Stage**	
Stage I	279 (56)
Stage II	52 (11)
Stage III	111 (22)
Stage IV	53 (11)
**TTN mutation**	
Wild-type	485 (98)
Mutate-type	10 (2)

### TTN mutations and biological characteristics

**Figure 2:**
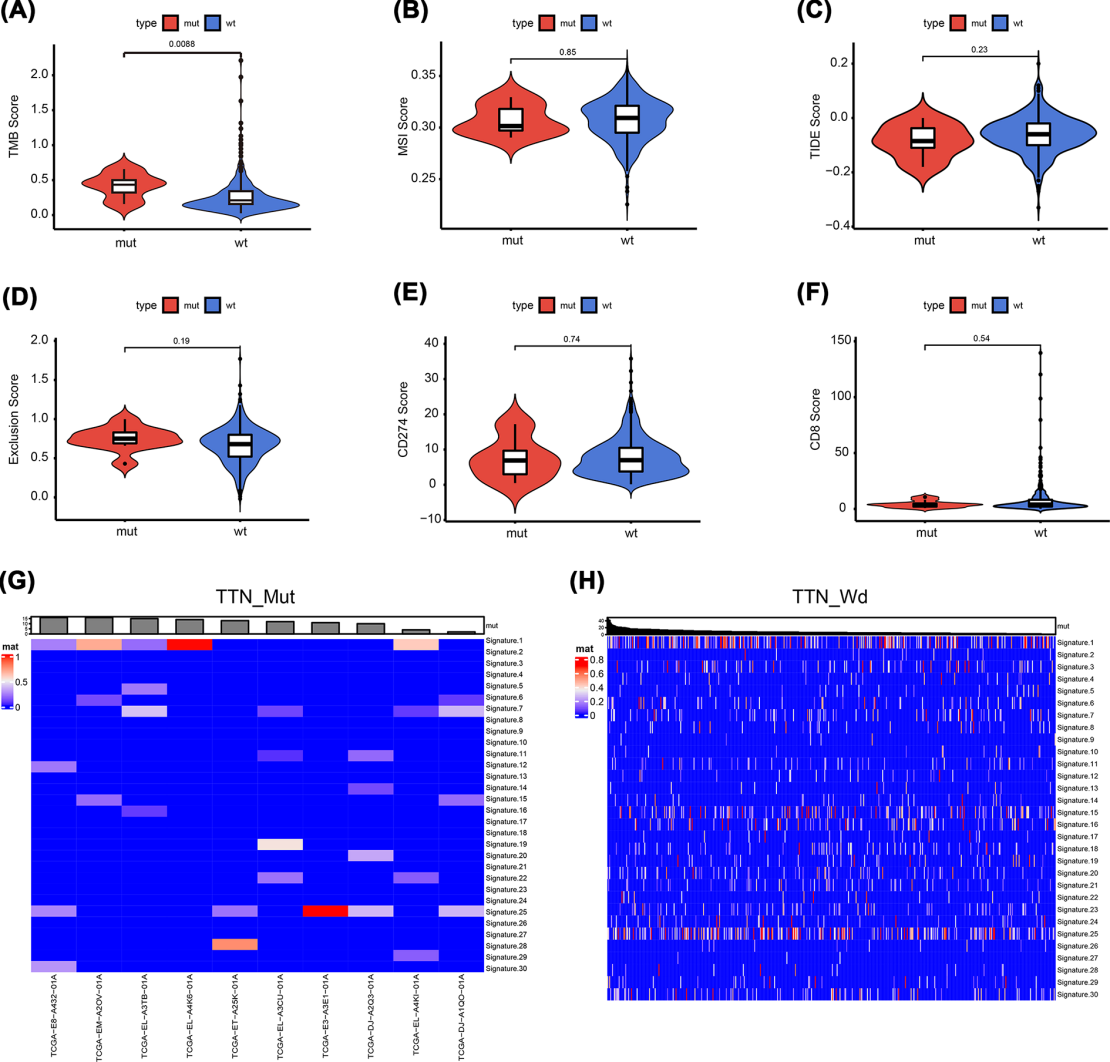
Analysis of biological characteristics of TTN mutations in patients with THCA (**A**) TMB levels were significantly increased in the MUT group (*P*<0.001). (**B–F**) No significant differences in MSI, TIDE score, immune escape score, CD274 and CD8 scores between the two groups. (**G,H**) Heat map analysis between the two groups.

We further analyzed the correlation between TTN gene mutations and different biological characteristics. The results showed that TMB levels were significantly increased in the MUT group compared with the WT *group (P*=0.0088), but there were no significant differences in microsatellite instability (MSI), TIDE score, immune escape score, CD274 score, or CD8 score between the two groups (Figure 2A–F). In addition, the spectrum of 96 mutations was decomposed into 30 different signatures and combined with biological characteristics and somatic mutation characteristics. The results showed that there was no significant change in signature distribution between the two groups (Figure 2G,H).

### Differential gene expression analysis in patients with TTN mutations

**Figure 3:**
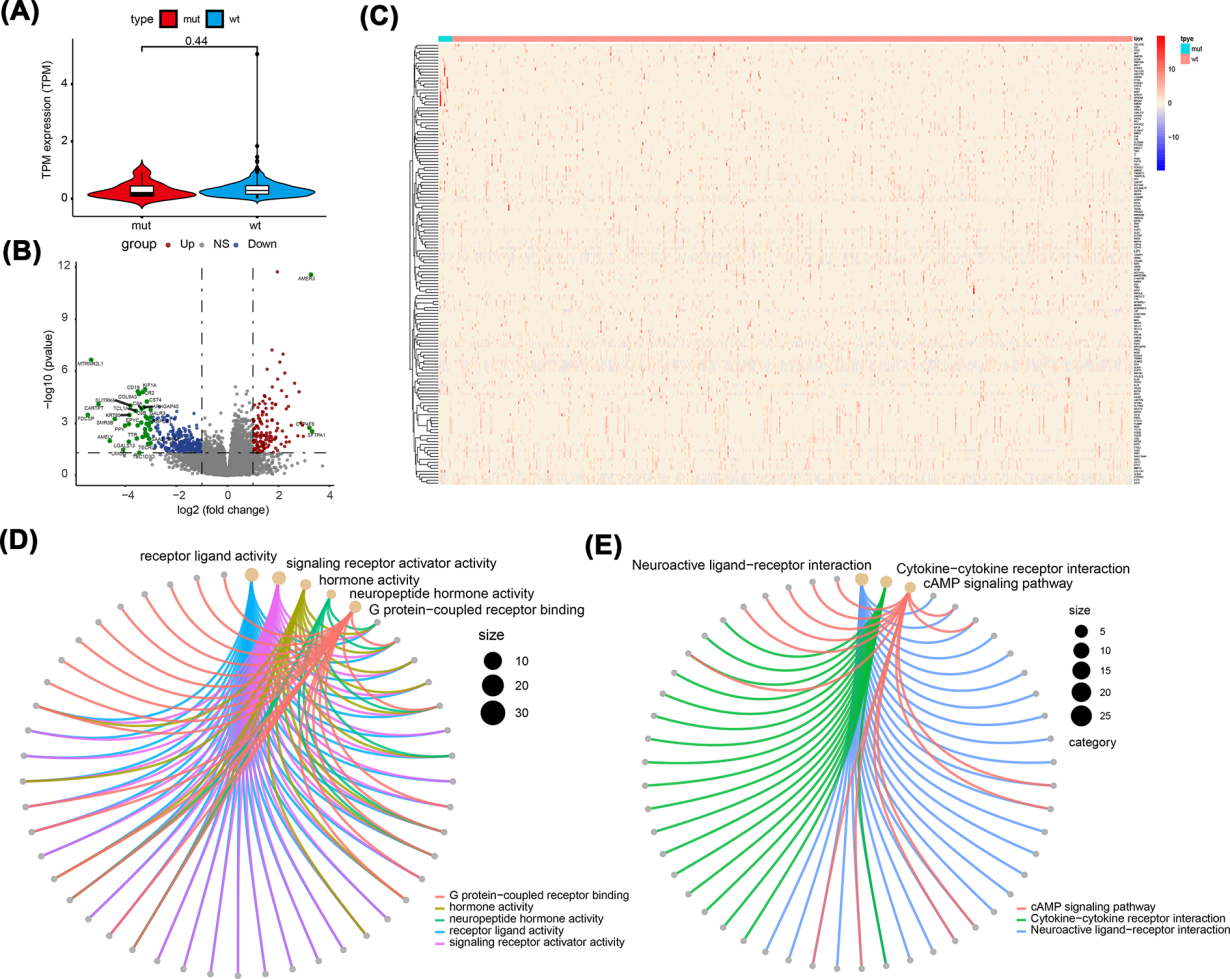
Differential expression analysis and functional enrichment analysis (**A**) TTN gene expression level between the two groups (*P*=0.053). (**B,C**) The volcano map and heat map between the two groups. (**D**) GO enrichment analysis. (**E**) KEGG pathway enrichment analysis.

To analyze the effect of TTN gene mutations on the development of THCA, samples with THCA from the TCGA database were divided into the MUT group and the WT group. TTN gene expression levels are shown in Figure 3A. Subsequently, differential expression analysis was conducted between the two groups. The results showed that 409 genes were significantly up-regulated in the MUT group, and 36 genes were significantly down-regulated in the MUT group (Figure 3B,C). Next, we performed functional enrichment analysis of the DEGs. GO enrichment analysis showed that the DEGs were closely related to the biological processes of receptor ligand activity, signalling receptor activator activity, and hormone activity (Figure 3D). KEGG pathway enrichment analysis suggested that the DEGs mainly affected neuroactive ligand–receptor interactions, cytokine–cytokine receptor interactions, and cAMP signalling pathways (Figure 3E).

**Figure 4:**
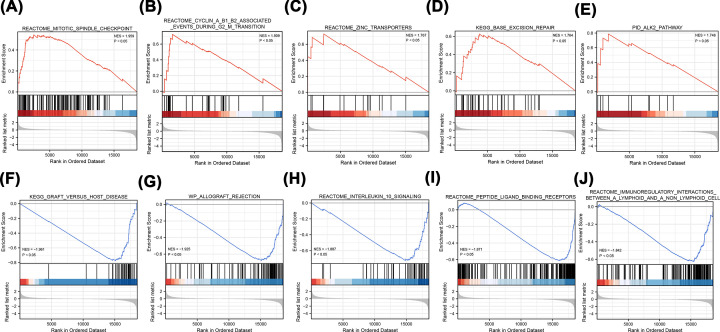
GSEA enrichment analysis (**A–D**) Based on NES values, the top five enriched pathways in the MUT group were shown. (**E–J**) Based on NES values, the top five enriched pathways in the WT group were shown. NES value represents the enrichment score after normalization. The higher the NES value, the more genes were enriched in the pathway.

Meanwhile, based on the gene expression profile in the TCGA-THCA dataset, GSEA enrichment analysis results showed that mitotic spindle checkpoint-, cyclin A/B1/B2-associated events during the g2 m transition, zinc transporters, and KEGG base excision repair pathways were significantly enriched in the MUT group. KEGG analysis showed that genes involved in graft versus host disease, allograft rejection, and interleukin-10 signalling and peptide ligand-binding receptor genes were significantly enriched in the WT group (Figure 4A–J).

### PPI and hub gene identification

**Figure 5:**
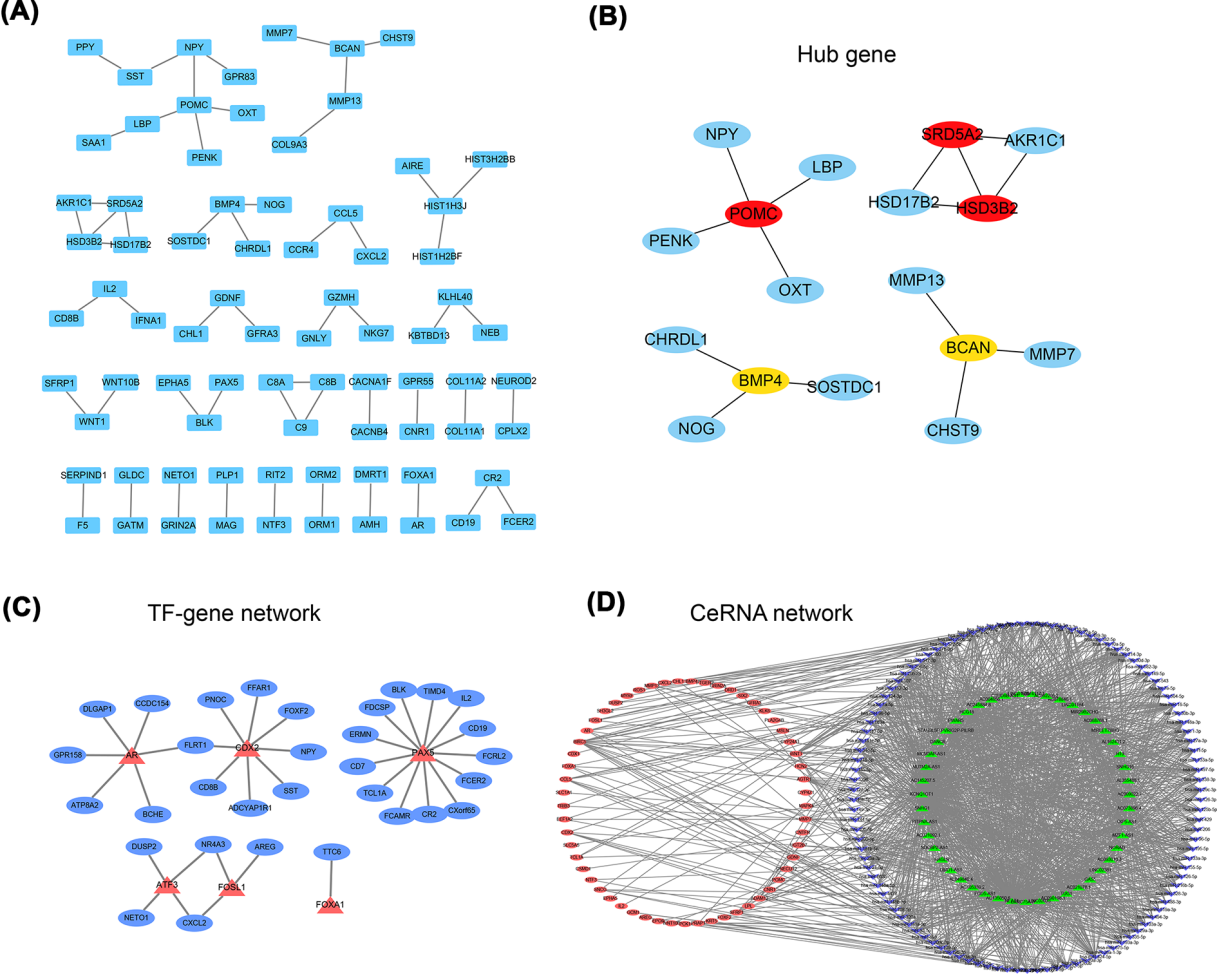
Construction of PPI, TF-target interaction networks, and ceRNA regulatory networks (**A**) PPI networks between DEGs was established through STRING database. Each node represents a different gene. (**B**) Hub gene. (**C**) TF-target interaction networks. Red represents TF and blue represents target genes. (**D**) ceRNA regulatory networks. Green, blue, and red represent lncRNA, miRNA, and mRNA, respectively.

A PPI network between DEGs was established through the STRING database (Figure 5A). Through the CytoHubba plug-in in Cytoscape, the MCC algorithm was used to select the top ten genes, which were called hub genes (Figure 5B). TF-target interaction networks and ceRNA regulatory networks were obtained from the Cistrome database and miRTarBase database (Figure 5C,D), respectively. These data were visualized using Cytoscape software.

### Effect of TTN mutations on immune cell infiltration in patients with THCA

**Figure 6:**
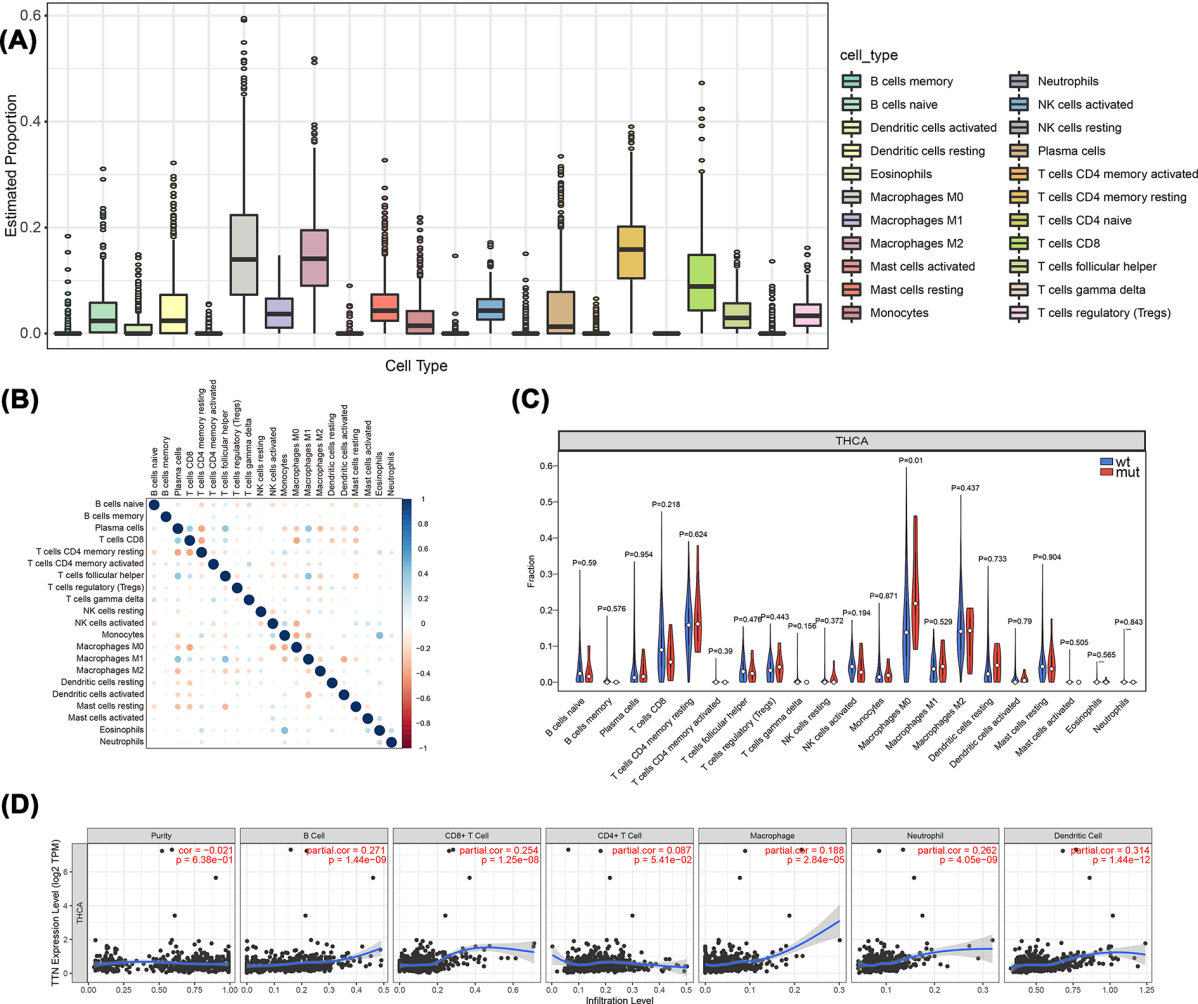
The relationship between TTN mutations and immune cell invasion (**A**) The panorama of immune cell infiltration. (**B**) Correlation analysis of 22 kinds of immune cells in the TCGA-THCA dataset. (**C**) Comparison of immune cell infiltration between the two groups. (**D**) Correlation analysis between TTN and different immune cells.

To analyze the relationship between TTN gene mutations and immune cell invasion in the tumor microenvironment, we calculated the proportion of immune cell invasion in the tumor microenvironment using the CIBERSORT algorithm. Figure 6A,B shows the panorama of immune cell infiltration and immune cell score, respectively. Then, the expression differences of immune cells in the two groups were further analyzed, and M0 macrophages were found to have a higher infiltration score in the MUT group than in the WT group (Figure 6C). Correlation analysis based on the TIMER database showed that there were significant differences in the infiltration levels of various immune cells among different TTN gene mutation levels (Figure 6D).

### Correlation analysis of TTN mutations and clinical features

**Figure 7:**
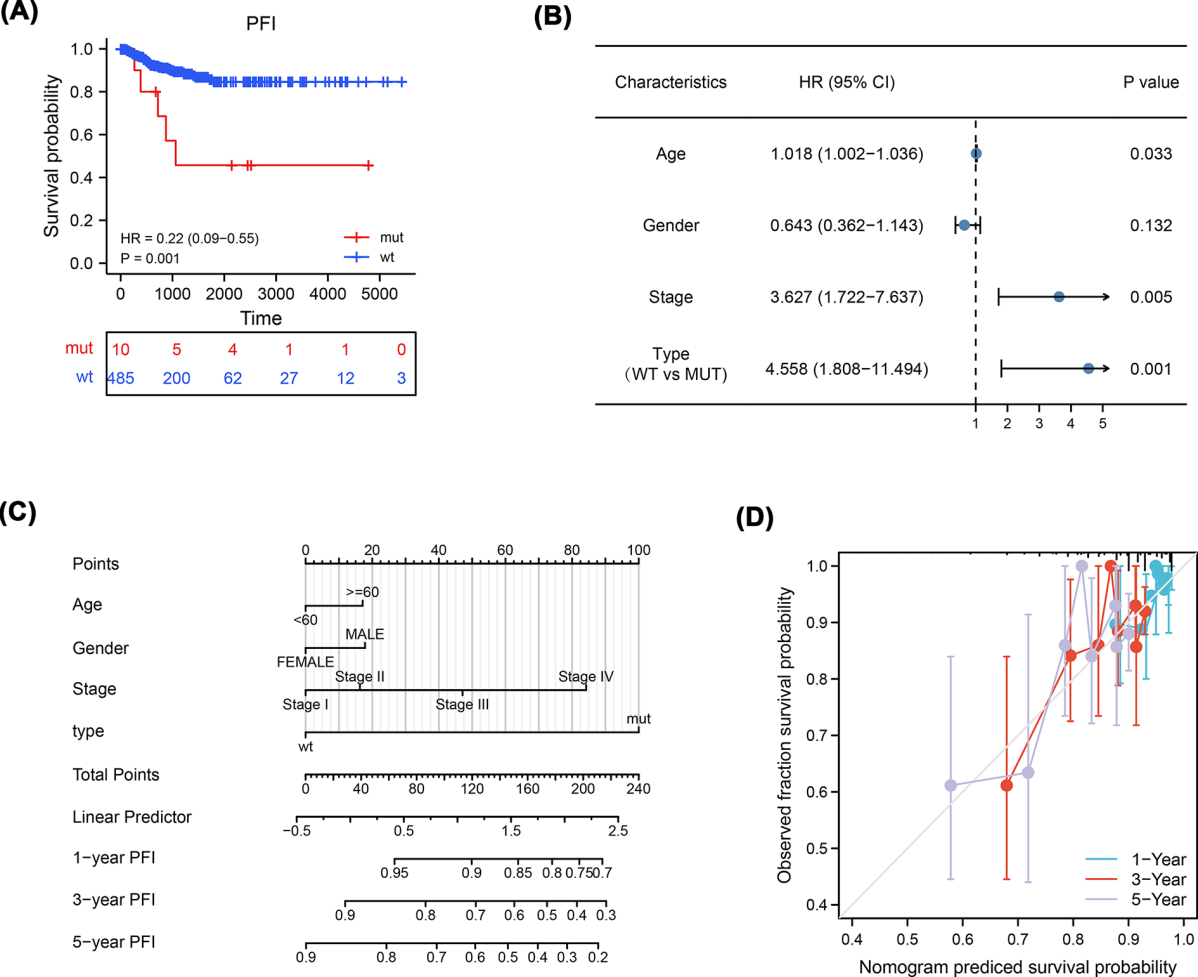
Effect of TTN mutations on clinicopathological features (**A**) Survival analysis showed that the MUT group predicted poorer prognosis (PFI, HR=0.22, [95% CI, 0.09–0.55], *P*=0.001) than the WT group. (**B**) Cox regression analyses demonstrated that TTN gene mutations were an independent risk factor for THCA (OS, HR=4.558, [CI, 1.808–11.494], *P*=0.001). (**C**) Construction of a nomogram. (**D**) Calibration curve of the nomogram.

To explore the association between TTN gene mutations and the clinical prognosis of patients with THCA, we conducted a clinical correlation analysis combining clinicopathological features. Survival analysis showed that the MUT group predicted poorer prognosis [progression-free interval (PFI), hazard ratio (HR)=0.22, [95% confidence interval (CI), 0.09–0.55], *P*=0.001] than the WT group (Figure 7A). Univariate and multivariate Cox regression analyses demonstrated that TTN gene mutations were an independent risk factor for THCA [overall survival (OS), HR=4.558, [CI, 1.808–11.494], *P*=0.001] (Figure 7B). Moreover, we combined TTN gene mutations with clinicopathological features to construct a nomogram to predict the prognosis of patients with THCA (Figure 7C). The efficiency of the nomogram was verified using the calibration curve, and the results showed that the 1-year nomogram had good consistency with the actual observed OS of patients (Figure 7D).

### TTN mutations and drug sensitivity

**Figure 8:**
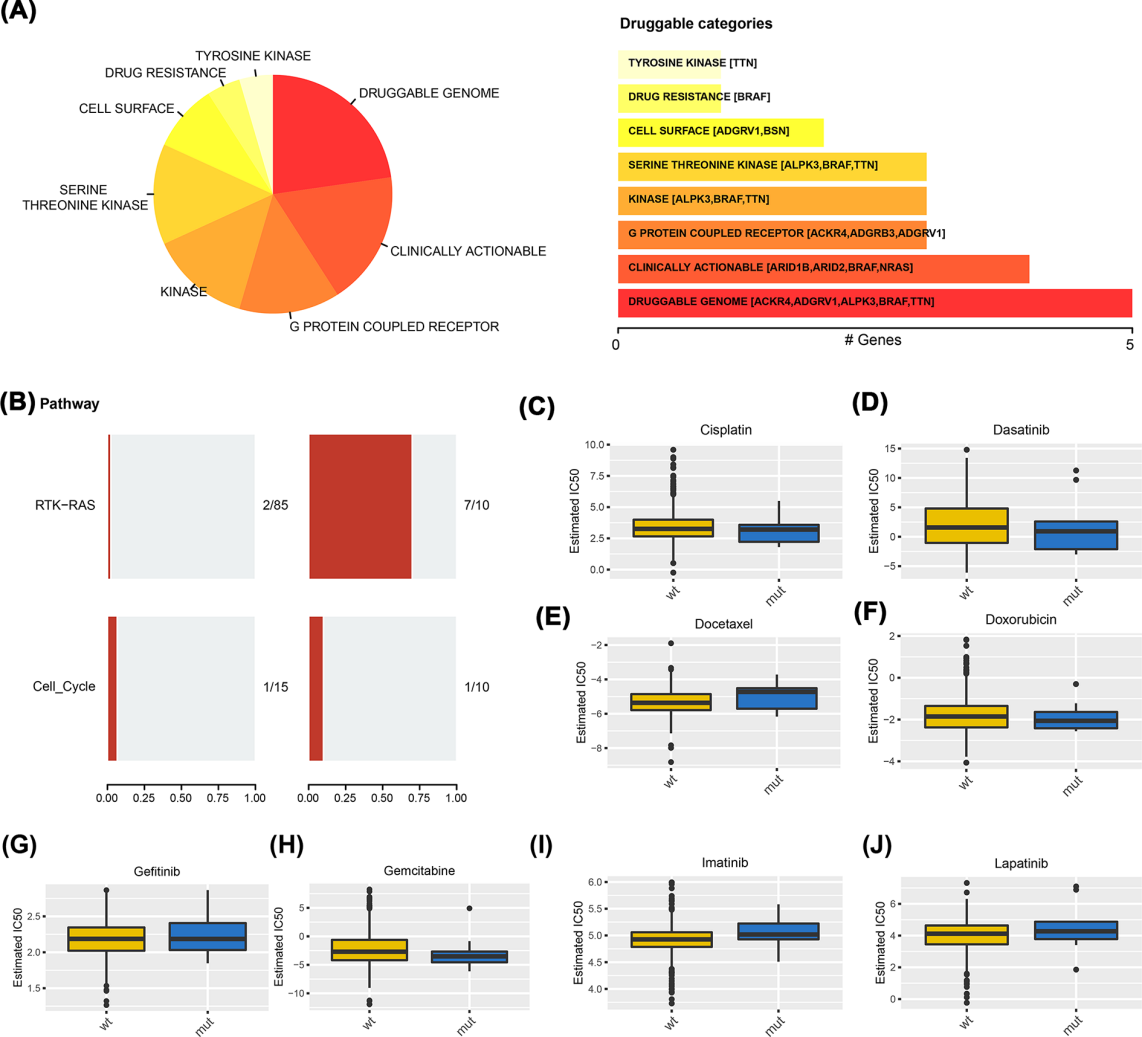
The relationship between TTN mutations and drug sensitivity (**A**) A variety of drugs were correlated with the level of TTN mutations. (**B**) Analysis of changes in gene mutation levels in different oncogenic signaling pathways. (**C–J**) IC50 values of antitumor drugs were analyzed by GDSC database between the two groups.

Finally, we analyzed the effect of TTN gene mutations on the drug sensitivity of patients with THCA and found that a variety of drugs were correlated with the level of TTN gene mutations, especially the druggable genome (Figure 8A). Pathway analysis showed that the RTK-RAS pathway was enriched in the MUT group (Figure 8B). Moreover, the IC50 values of antitumor drugs between the MUT group and WT group were analyzed using the GDSC database, and the results showed that cisplatin, docetaxel, and imatinib had different trends (Figure 8C–J).

## Discussion

Many molecular alterations represent novel diagnostic and prognostic molecular markers and therapeutic targets for cancer, which provide unprecedented opportunities for further research and clinical development of novel treatment strategies [38]. In recent years, studies have shown that TTN gene mutations may be involved in the development of tumors. TTN is the largest described protein and constitutes the third most abundant type of filament in both cardiac and skeletal human muscle, together with actin and myosin [39]. The entire TTN gene consists of 364 exons and is located on chromosome 2q31 [40]. Single-cell DNA sequencing revealed that the most common mutation in gastroesophageal carcinoma is the TTN gene, which was reported to be associated with TMB in cancer [41]. TTN mutations play an important role in human cancer. However, the relationship between TTN mutations and THCA has not been reported. Hence, we performed a comprehensive bioinformatics analysis of TTN gene mutations and survival prognostic value in THCA. Our results demonstrated that TTN gene mutations predict poor prognosis in patients with THCA and that the TTN gene may serve as a potential prognostic indicator of THCA. This is the first study to research TTN gene mutations in THCA and to investigate their prognostic value in THCA.

TTN mutations associated with human conditions include nonsense mutations, frameshift mutations, missense mutations, and splice site mutations. Studies have reported that TTN missense mutations are correlated with lung squamous cell carcinoma [42,43]. Our study also proved that missense mutations account for the dominant mutations in patients with THCA. Next, to analyze the effect of TTN gene mutations on tumorigenesis in THCA, we divided the patients with THCA into the MUT group and the WT group. Differential expression analysis showed that 409 genes were significantly up-regulated and 36 genes were down-regulated in the MUT group compared with the WT group. Moreover, functional enrichment analysis was performed for the DEGs, including GO enrichment analysis, KEGG pathway enrichment analysis, and GSEA. Functional enrichment analysis revealed that TTN gene mutations played a potential role in the development of THCA. In addition, a PPI network was established. The results showed that TTN gene mutations mainly interact with POMC, SRD5A2, HSD3B2, BMP4, and BCAN. It was recently reported that these genes are involved in the progression of cancer [44–46]. We also explored the molecular mechanism of TTN gene mutations in THCA through TF-target interaction networks and ceRNA regulatory networks.

Increasing evidence has shown that immune infiltration is closely related to cancer [47–49]. Novel-targeted drugs and immunotherapies have developed rapidly over the last decade, bringing new treatments to patients with malignant tumors. Hence, we further analyzed the relationship between the carcinogenic effect of TTN gene mutations and immune infiltration. Analysis of the THCA immune microenvironment indicated that TTN mutation was significantly associated with enrichment of M0 macrophages (*P*<0.05).

Interestingly, we focused on the clinical significance of TTN gene mutations in THCA and discovered that TTN gene mutations may be a potential prognostic indicator for patients with THCA. First, survival analysis showed that the MUT group had a poorer predicted prognosis (PFI, HR=0.22, [95% CI, 0.09–0.55], *P*=0.001) than the WT group. Then, Cox regression analysis demonstrated that TTN gene mutations were an independent risk factor for THCA (OS, HR=4.558, [95% CI, 1.808–11.494], *P*=0.001). More importantly, we combined TTN gene mutations with clinicopathological features to construct a nomogram. The nomogram also confirmed the prognostic value of the TTN gene in THCA. In addition, TTN mutations resulted in increased thyroid cancer sensitivity to eight drugs (*P*<0.05), especially the druggable genome. These results suggest that the TTN gene plays a unique and crucial role in the prognosis of THCA.

Nevertheless, there are some limitations to our study. First, although bioinformatics analysis is a powerful and efficient tool to aid in understanding of molecular mechanisms and for identifying potential biomarkers underlying THCA, further experimental validations of the TTN gene are needed at the molecular, cellular, and organismal levels. Second, the present study focused on the prognostic value rather than the predictive value of TTN mutations in patients with THCA. Finally, further experimental evidence, such as evidence obtained from real-time PCR, western blot, and immunohistochemistry assays, is required to fully elucidate the role of hub genes and the underlying mechanisms of THCA.

In conclusion, further exploration of the potential value of the TTN gene as a therapeutic target is highly warranted and may ultimately lead to the development of innovative drug therapies for complex and serious diseases. This is the first study to demonstrate TTN gene mutations in THCA and to investigate their prognostic value in THCA. The present study will provide a potential therapeutic strategy for thyroid cancers.

## Conclusions

In summary, new gene mutations in THCA have been identified through bioinformatics analysis, and these results contribute to the treatment and prognosis of thyroid cancer. This is the first study to demonstrate TTN gene mutations in THCA and to investigate their prognostic value in THCA.

## Supplementary Material

Supplementary Figure S1

## Data Availability

Our study used public online database. The data can be accessed by following websites: https://cloud.genepattern.org, https://www.gsea-msigdb.org, http://geneontology.org/, http://www.genome.jp/kegg, https://string-db.org/, http://www.cistrome.org/, https://mirtarbase.cuhk.edu.cn/, and http://www.cancerRxgene.org.

## Abbreviations

ATC: anaplastic thyroid cancer
BP: biological process
CC: cellular component
ceRNA: competitive endogenous RNA
CI: confidence interval
CNV: copy number variation
DEG: differentially expressed gene
FDR: false discovery rate
FTC: follicular thyroid cancer
GDSC: Genomics of Drug Sensitivity in Cancer
GO: genetic ontology
GSEA: gene set enrichment analysis
HR: hazard ratio
KEGG: Kyoto Encyclopedia of Genes and Genomes
MCC: maximal clique centrality
MF: molecular function
MSI: microsatellite instability
MUT: mutant-type
OS: overall survival
OSSN: ocular surface squamous neoplasia
PDTC: poorly differentiated thyroid cancer
PFI: progression-free interval
PPI: protein–protein interaction
PTC: papillary thyroid cancer
RAI: radioactive iodine
SNV: single-nucleotide variant
TF: transcription factor
THCA: thyroid cancer
TMB: tumor mutation burden
TSH: thyroid stimulating hormone
TTN: titin
WT: wild-type
