# Supplementary material for: TTN mutations predict a poor prognosis in patients with thyroid cancer
Source: Biosci Rep. 2022 Jul 22;42(7):BSR20221168. doi: 10.1042/BSR20221168 (PMC9310696; doi:10.1042/BSR20221168)
Supplement: Supplementary Figure S1 [file BSR-2022-1168_supp.pdf]

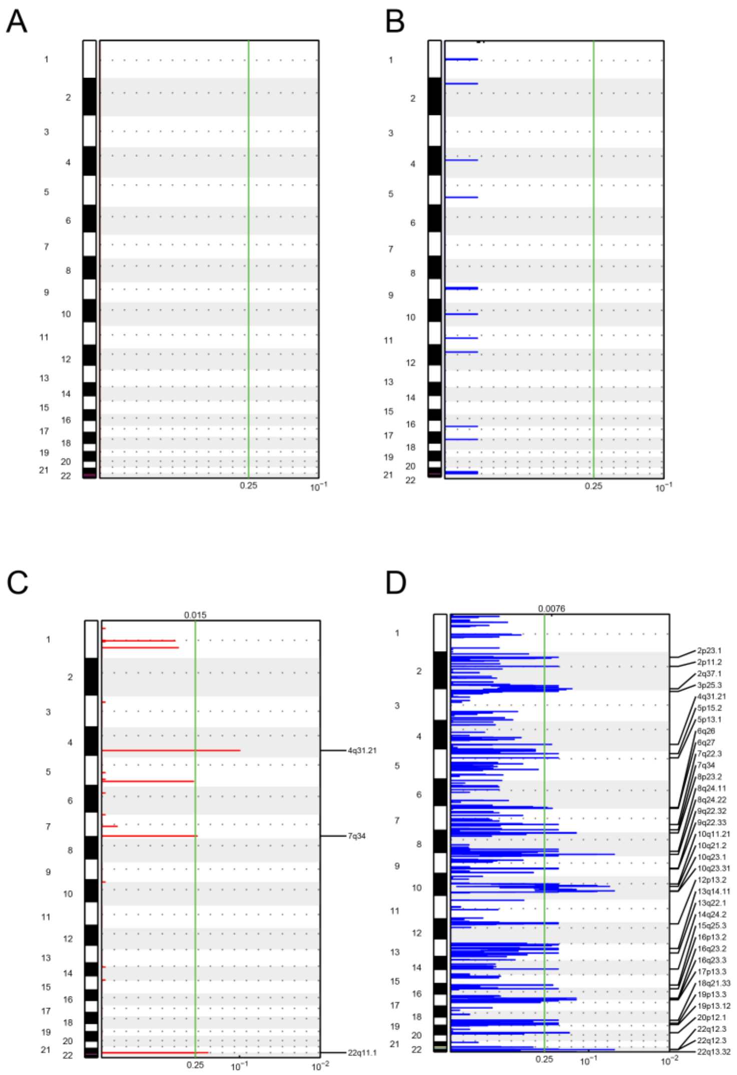

Figure S1 Copy Number Variation in Patients with THCA. (A-B) Copy number amplification and copy number deletion in the MUT group. (C-D) Copy number amplification and copy number deletion in the WT group. Red represents amplification and blue represents deletion.
